# Lung ultrasound presentation of COVID-19 patients: phenotypes and correlations

**DOI:** 10.1007/s11739-020-02620-9

**Published:** 2021-03-01

**Authors:** Gianmarco Secco, Marzia Delorenzo, Francesco Salinaro, Caterina Zattera, Bruno Barcella, Flavia Resta, Anna Sabena, Giulia Vezzoni, Marco Bonzano, Federica Briganti, Giovanni Cappa, Francesca Zugnoni, Lorenzo Demitry, Francesco Mojoli, Fausto Baldanti, Raffaele Bruno, Stefano Perlini, The GERICO (Gruppo Esteso RIcerca COronarovirus) Lung US Pavia Study Group, Ilaria Martino, Barbarah Guglielmana, Ilaria Zunino, Federica Quaglia, Pietro Pettenazza, Serena Pioli di Marco, Domenica Federica Briganti, Anna Giulia Falchi, Claudia Alfano, Elisa Mossolani, Massimiliano Sciarrini, Caterina Zattera, Igor Maisak, Michele Tassi, Stefano Galati, Capozza, Ilaria Melara, Benedetta Chiodi, Damiano Vignaroli, Lorenzo Scattaglia, Giulia Bissichini, Marco Bazzini

**Affiliations:** 1 Emergency Medicine Unit and Emergency Medicine Postgraduate Training Program, Internal Medicine, Vascular and Metabolic Disease Unit, Department of Internal Medicine, IRCCS Policlinico San Matteo Foundation, University of Pavia, P.Le Golgi, 19, 27100 Pavia, Italy; 2 Intensive Care Unit, Department of Internal Medicine, IRCCS Policlinico San Matteo Foundation, University of Pavia, Pavia, Italy; 3 Virology Unit, Department of Internal Medicine, IRCCS Policlinico San Matteo Foundation, University of Pavia, Pavia, Italy; 4 Infectious Disease Unit, Department of Internal Medicine, IRCCS Policlinico San Matteo Foundation, University of Pavia, Pavia, Italy

**Keywords:** COVID-19, Lung ultrasound, Echographic phenotypes, LUS score, Interstitial pneumonia

## Abstract

Bedside lung ultrasound (LUS) can play a role in the setting of the SarsCoV2 pneumonia pandemic. To evaluate the clinical and LUS features of COVID-19 in the ED and their potential prognostic role, a cohort of laboratory-confirmed COVID-19 patients underwent LUS upon admission in the ED. LUS score was derived from 12 fields. A prevalent LUS pattern was assigned depending on the presence of interstitial syndrome only (Interstitial Pattern), or evidence of subpleural consolidations in at least two fields (Consolidation Pattern). The endpoint was 30-day mortality. The relationship between hemogasanalysis parameters and LUS score was also evaluated. Out of 312 patients, only 36 (11.5%) did not present lung involvment, as defined by LUS score < 1. The majority of patients were admitted either in a general ward (53.8%) or in intensive care unit (9.6%), whereas 106 patients (33.9%) were discharged from the ED. In-hospital mortality was 25.3%, and 30-day survival was 67.6%. A LUS score > 13 had a 77.2% sensitivity and a 71.5% specificity (AUC 0.814; *p* < 0.001) in predicting mortality. LUS alterations were more frequent (64%) in the posterior lower fields. LUS score was related with P/F (*R*^2^ 0.68; *p* < 0.0001) and P/F at FiO_2_ = 21% (*R*^2^ 0.59; *p* < 0.0001). The correlation between LUS score and P/F was not influenced by the prevalent ultrasound pattern. LUS represents an effective tool in both defining diagnosis and stratifying prognosis of COVID-19 pneumonia. The correlation between LUS and hemogasanalysis parameters underscores its role in evaluating lung structure and function.

## Introduction

At the beginning of December 2019, an outbreak of pneumonia cases with a viral-like clinical presentation took place in Wuhan, Hubei province, China. The pathogen isolated was named SARS-CoV-2, being responsible of Coronavirus Disease-19 (COVID-19) [1, 2] The first indigenous case of COVID 19 in Italy was confirmed on February 20, 2020 in Codogno (Lodi), and the last update on COVID-19 Global Cases by John Hopkins CSSE on May 27th reported 5.609.079 confirmed cases, with 350.862 deaths worldwide [3]. The SARS-CoV-2 infection can generate different responses in patients, ranging from completely asymptomatic virus shedding to a severe inflammatory response including cytokine storm-like outcomes that is accompanied by high mortality [4]. However, as suggested by Gattinoni and coworkers [5], COVID-19 pneumonia is a specific disease with peculiar phenotypes, although it can satisfy the ARDS Berlin definition [6]. In detail, these Authors point out that “its main characteristic is the dissociation between the severity of the hypoxemia and the maintenance of relatively good respiratory mechanics”, and propose two different clinical presentations with a distinct physiopathology [7, 8]. In detail, type L phenotype (also defined as “non-ARDS” pattern) is characterised by low elastance (i.e., high compliance), low ventilation-to-perfusion ratio, low lung weight and low recruitability, whereas Type H phenotype presents with high elastance, high right-to-left shunt, high lung weight and high recruitability (hence the definition of “ARDS” pattern) [5, 7, 8]. Of note, the extent of hypoxemia is similar in patients with respiratory compliance lower or higher than the median value [5]. The two patterns can be distinguished either by CT scan evaluation or by respiratory system compliance and the response to PEEP. In Type L patients, imaging shows only ground-glass densities, primarily located in the subpleural regions and along the lung fissures, whereas in Type H quantitative analysis of the CT scan shows marked increase in lung weight, with bilateral infiltrates [7].

An alternative imaging technique might be represented by bedside lung ultrasound (LUS) examination, and LUS estimates of the ratio between tissue and air on the superficial lung have been shown to correlate with tissue density on quantitative CT, and with different parameters of lung dysfunction, such as P/F ratio, in patients with ARDS [9, 10] as well as in influenza A (H1N1) viral infection [11, 12]. Indeed, tissue/air ratio in the superficial lung causes different LUS presentations, ranging from localized vertical artifacts to progressive coalescence in a homogeneous hyperechoic picture named “white lung”, to parenchymal consolidations [13].

Among the many critical challenges posed by the current COVID-19 outbreak to the clinician, one diagnostic dilemma is represented by the need of rapidly identifying this new form of pneumonia. In this setting, LUS has the advantage of being performed at the bedside concomitant to the clinical evaluation of the patient, when waiting for the diagnostic confirmation by the nasopharyngeal swab. Aim of the present paper was to evaluate in a cohort of consecutive COVID-19 patients presenting in the Emergency Department (ED) the clinical and ultrasound features of the disease, with special attention between LUS findings and arterial blood gas evaluation. The potential prognostic role of LUS in this setting was assessed having mortality as a primary outcome, censored at 30 days.

## Materials and methods

**Fig. 1:**
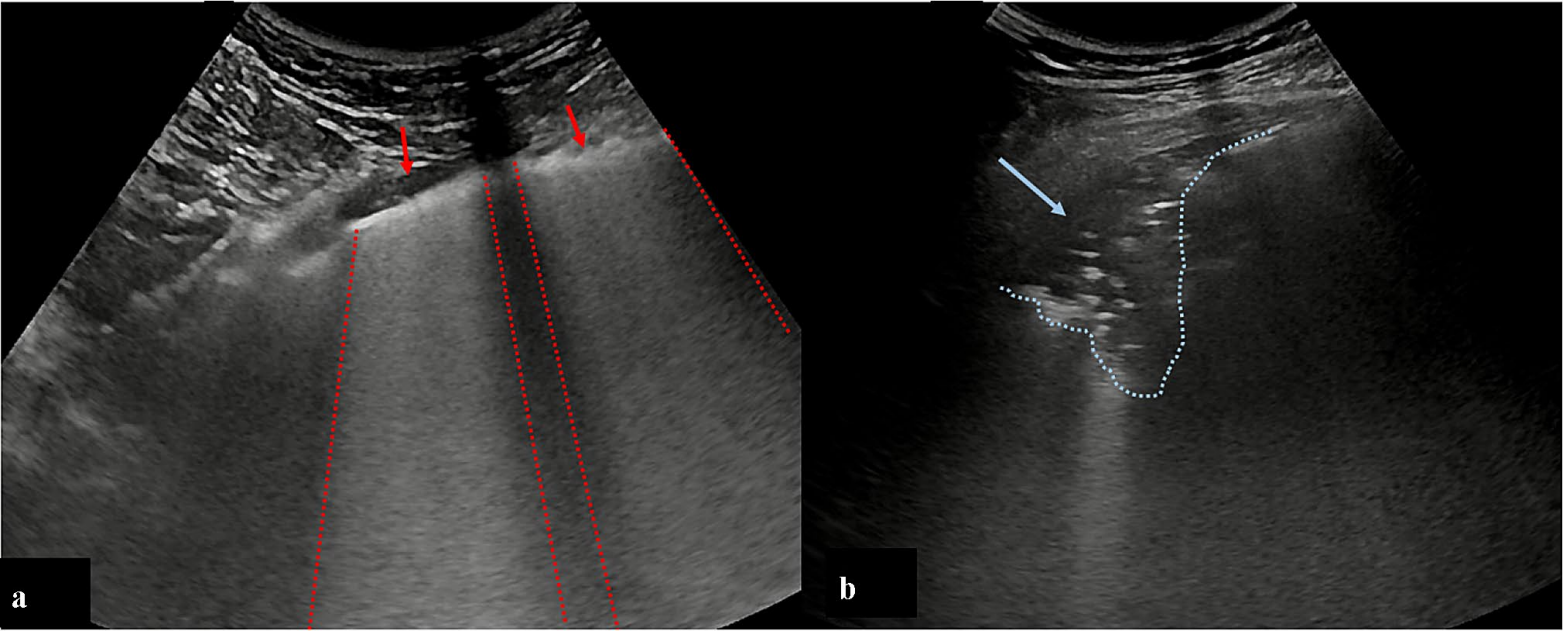
**a** Red arrows indicate areas of interstitial syndrome (interstitial pattern); **b** arrow indicates a subpleural consolidation (cosolidative pattern)

The study enrolled consecutive patients with laboratory-confirmed COVID-19, from March 2nd to April 22nd, 2020. A positive result on high throughput sequencing or real-time reverse-transcriptase–polymerase-chain-reaction (RT-PCR) assay of nasal and pharyngeal swab was the fundamental requirement to be included in the final analysis. After having obtained written informed consent, all patients underwent lung ultrasound, associated with a pre-specified “suspected COVID-19” laboratory test profile, including complete blood count, assessment of renal and liver function, Troponin I (TnI), serum electrolytes, C-reactive protein (CRP), lactate dehydrogenase (LDH), and creatine kinase (CPK). Upon ED admission, vital parameters, body temperature, arterial blood gas parameters and presentation symptoms were collected. Per protocol, while waiting for the swab results, all patients underwent bedside LUS evaluation with Aloka Arietta V70 (Hitachi Medical Systems, Buccinasco, Milano, Italy), equipped with a convex 5 MHz probe. The image acquiring procedure was standardized using the abdominal set, maximum 10 cm depth, focus on the pleural line. Gain was adjusted to obtain the best possible image of the pleura, vertical artifacts and peripheral consolidations with or without air bronchograms. All harmonics and artifact-erasing software were disabled. Both longitudinal and transversal scans were performed to explore a wider and larger pleural length [14]. Thorax was studied with the patient in the supine or semi-supine position, depending on the level of cooperation. According to guidelines in the emergency setting [15], LUS examination was conducted by trained ED physicians (experienced sonographers on the basis of the American College of Emergency Physicians ultrasonographic guidelines and more than 10 ultrasound exams performed per week, 5 years of experience in performing and interpreting POCUS) [16], using 12 windows (2 anterior, 2 lateral and 2 posterior zones per hemithorax). The anterior zones were imaged from the parasternal line to the anterior axillary line, and the two lateral zones between the axillary anterior and posterior lines. Superior and inferior areas were divided by the intermammary line (the superior 2–3 spaces as the superior chest and the other 2–3 spaces the inferior chest area).[17] Videoclips were recorded, ensuring analysis throughout the respiratory cycle, to allow subsequent off-line re-evaluation. Special attention was given to the topography of vertical artifacts, the gradient of distribution and the regularity/irregularity of pleural line, the presence of pleural effusion. In each region, a quantitative LUS score was attributed by an external reader, who was blinded to the clinical presentation, as follows: score 0: normal lung aeration (A lines or less than two small vertical artifacts); score 1: 1 mild loss of aeration (presence of vertical artifacts either lung consolidation in less than 50% of the pleural line); score 2: severe loss of aeration (“white lung”or coalescent B vertical artifacts or presence of vertical artifacts/lung subpleural consolidation in more than 50% of the pleural line); score 3: complete loss of aeration (predominant tissue-like pattern) [9, 18]. Global LUS score was computed as the sum of each regional scores. A prevalent LUS pattern was assigned depending on the presence of only interstitial syndrome (“Interstitial Pattern”), or evidence of subpleural consolidations in at least two lung fields (“Consolidation Pattern”), in which the presence of vertical artifacts also coexisted. (Fig. 1). The absence of lung injury was defined as a LUS score < 1. The association between 30-day mortality and LUS findings upon ED admission (both LUS score and prevalent LUS pattern) was assessed. The relationship between LUS score and respiratory arterial blood gas parameters was evaluated in the whole group as well as in the two different LUS patterns. For the statistical analysis, the software MEDCALC 19.2.3 version for Windows was used. Continuous variables were expressed as median values, while categorical variables were expressed as percentage. A *p* < 0.05 value was considered statistically significant. Scatter diagrams, ANOVA, regressions, Kaplan–Meier curves and Receptor Operating Characteristic (ROC) curve and χ2 analyses were used, as appropriate. Multivariable analysis was performed to assess whether LUS score was a predictor of 30-day mortality independent of lymphocyte count, LDH, aPTT and white count. No imputation was made for missing data. Because the cohort of patients in our study was not derived from random selection, all statistics are deemed to be descriptive only. The prognosis was censored at 30 days through medical records for hospitalised patients and through phone calls for discharged subjects.

## Results

### Patient characteristics

From March 2nd to April 22nd, 2020 we evaluated 820 patients with flu-like symptoms, suspected for COVID-19. The first 312 (on 523) with a positive nasal swab for Sars-Cov-2 constituted our study cohort. The general features of these patients with laboratory-confirmed COVID-19 are shown in Table 1, 2, and 3. Median age was 64 years (range: 22–94), with a 66.3% male prevalence. The two most frequent symptoms were fever (> 37.5 °C; 88.5%) and dyspnoea (53.5%). Hypertension was the more common comorbidity (50.6%). When compared with survivors, dead patients had a significant higher number of comorbities (2 vs 1, *p* < 0.001). As expected, lymphocyte count was reduced (0.9; 0.1–3.9) whereas CRP (7.6 mg/dL; 0.01–43.9) and LDH (329 mU/mL; 122–2578) were increased. Out of these 312 patients, 106 (34.0%) were discharged from the ED and confined to home quarantine, 168 (53.8%) were admitted in a general medical ward, 30 needed admission to ICU (9.6%), and 8 (2.8%) died in the ED. In-hospital mortality was 25.3% (8 patients in ED, 50 in medical ward, 21 in ICU). At the 30-day endpoint, global survival was 67.6%.

**Table 1:** Main features of the study population

	Overall (*n* = 312)	Discharged (*n* = 106)	Admitted. general ward (GW) (*n* = 168)	Admitted. intensive care unit (ICU) (*n* = 30)	Died in ER (*n* = 8)	*P* value (< 0.05)
Age (years)	64 (22–94)	52.5 (22–88)	71 (25–94)	64 (38–75)	83 (57–90)	*#§$
Sex (male %)	66.3	53.8	70.8	86	66.7	*#§$
BMI (kg/m^2^)	26.2 (18.7–45.7)	25 (18.7–45.7)	26.5 (19–40.8)	28 (20.8–39.2)	30.6 (7.3–33.8)	
Arterial systolic pressure (mmHg)	130 (80–190)	130 (90–185)	130 (89–190)	135 (80–178)	120 (85–160)	
Arterial diastolic pressure (mmHg)	80 (50–120)	80 (60–117)	80 (50–120)	80 (50–102)	80 (55–110)	
Heart rate (bpm)	89 (40–175)	88 (40–130)	89 (50–175)	95 (55–120)	85 (71–140)	
Respiratory rate (/min)	20 (10–75)	18 (10–44)	20 (10–50)	20 (10–37)	29 (20–75)	
CRP (mg/dL)	7.6 (0.01–43.9)	2.2 (0.01–15.7)	11.5 (0.04–44)	17.9 (0.6–40.2)	13.4 (2.6–35.7)	*#§$°
Hb (g/dL)	13.9 (4.6–23.5)	13.9 (10.1–17.1)	13.7 (4.6–23.5)	14.5 (10.5–16.6)	13.5 (10.3–16.3)	
Lymphocytes (× 10^3^/µl)	0.9 (0.1–3.9)	1.1 (0.4–2.9)	0.8 (0.1–3.9)	0.7 (0.2–1.4)	0.8 (0.5–1.1)	#§
LDH (mU/mL)	329 (122–2578)	241 (122–501)	370 (183–2578)	505 (215–1150)	462 (210–783)	#§
TnI (ng/mL)	11 (2.5–44783)	3 (2.5–125)	15 (2.5–44783)	14 (3–171)	27.5 (18–183)	
CPK (mU/mL)	130 (19–46737	93.5 (22–2130)	136 (19–46737)	256 (24–1041)	121 (34–698)	
Creatinine (mg/dL)	0.89 (0.37–17)	0.77 (0.37–1.69)	0.98 (0.44–4.3)	0.99 (0.56–1.6)	1.1 (0.7–17)	*$ˆ
PaO_2_/FiO_2_	306.7 (37.4–704.7)	380.5 (216.7–704.8)	261 (37.4–528.5)	201.9 (67.2–474.3)	174 (44.4–414.3)	*#§$
PaCO_2_ (mmHg)	32.7 (16.9–52)	34.7 (18.5–43)	32 (16.9–54.4)	32 (24–40)	35.1 (27.1–52)	§
PaO_2_ (mmHg)	71.3 (31–359)	80 (52–359)	66.3 (31–285)	61 (31.5–112)	67 (36.7–256)	#
LUS score	11 (0–25)	4 (0–20)	13 (0–25)	17 (8–24)	19 (6–24)	*#§°

General features of the study cohort of consecutive patients with laboratory-confirmed COVID-19. Data are shown as median value (range)

ANOVA (Shapiro–Wilk): * discharged vs dead; # discharged vs ICU; § discharged vs GW; $ dead vs IC; ° GW vs ICU; ˆ dead vs GW

**Table 2:** Comparison between dead and survived Covid-19 patients

	Dead (*n* = 79)	Survived (*n* = 165)	*p* value
Age (years)	77 (28–94)	60 (23–90)	*p* < 0.001
Sex (male %)	67	67.9	n.s
BMI (kg/m^2^)	27 (19.8–40)	26.1 (18.7–45.7)	n.s
Arterial systolic pressure (mmHg)	130 (80–178)	130 (89–190)	n.s
Arterial diastolic pressure (mmHg)	78 (50–120)	80 (50–117)	n.s
Heart rate (bpm)	87 (50–175)	90 (40–140)	n.s
Respiratory rate (/min)	24 (10–75)	20 (10–44)	*p* = 0.002
C-Reactive Protein (CRP) (mg/dL)	13.3 (0.5–43.9)	6.9 (0.01–38.2)	*p* < 0.001
Hemoglobyn (Hb) (g/dL)	13.2 (4.6–16.8)	14 (10–23.5)	*p* < 0.001
Lymphocytes (× 10^3/µL)	0.7 (0.1–2.5)	0.9 (0.2–2.9)	*p* = 0.001
Lactate dehydrogenase (LDH) (mg/dL)	422.5 (210–1235)	316 (122–2578)	*p* = 0.004
Troponin I (TnI) (ng/mL)	31 (2.5–44,783)	7 (2.5–5383)	n.s
Creatin kinase (CPK) (mU/mL)	142 (24–3140)	134 (24–46,737)	n.s
Creatinine (mg/dL)	1.1 (0.6–17)	0.86 (0.44–4.4)	*p* = 0.001
PaO_2_/FiO_2_	165 (37.3–414.3)	313 (74–704.7)	*p* < 0.001
PaCO2 (mmHg)	32.3 (16.9–59)	32.3 (18.5–42.2)	n.s
PaO2 (mmHg)	66 (31–256)	71.2 (38.2–352)	n.s
LUS score	18 (5–25)	10 (0–24)	*p* < 0.001

General features of the survived and dead (30 days) patients with laboratory-confirmed COVID-19. Data are shown as median value (range)

**Table 3:** Symptoms and comorbidities distribution in the study cohort

Symptoms		Comorbidities	
Fever	88.5%	COPD	4.8%
Dry cough	43.3%	Asthma	4.5%
Cough with sputum	2.9%	CAD	17.6%
Dyspnoea	53.5%	Hypertension	50.6%
Chest pain	7.1%	Diabetes	17%
Vomit	4.2%	Active cancer	4.5%
Diarrhea	9.6%	CKD	7.4%
Confusion	2.9%	Liver disease	2.9%
Asthenia	11.9%	Neurological disease	4.8%

Chronic obstructive pulmonary disease (COPD), coronary artery disease (CAD), chronic kidney disease (CKD)

### LUS findings

**Fig. 2:**
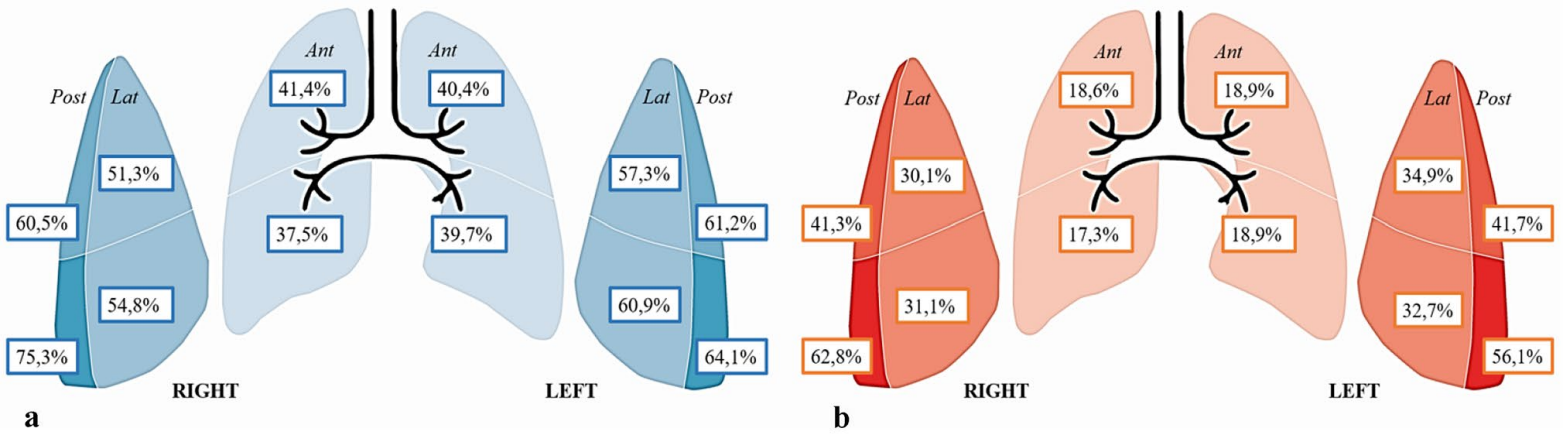
**a** Percentage distribution of both vertical artefacts and consolidations in the different lung fields; **b** percentage distribution of consolidation lesions in the different lung field

**Fig. 3:**
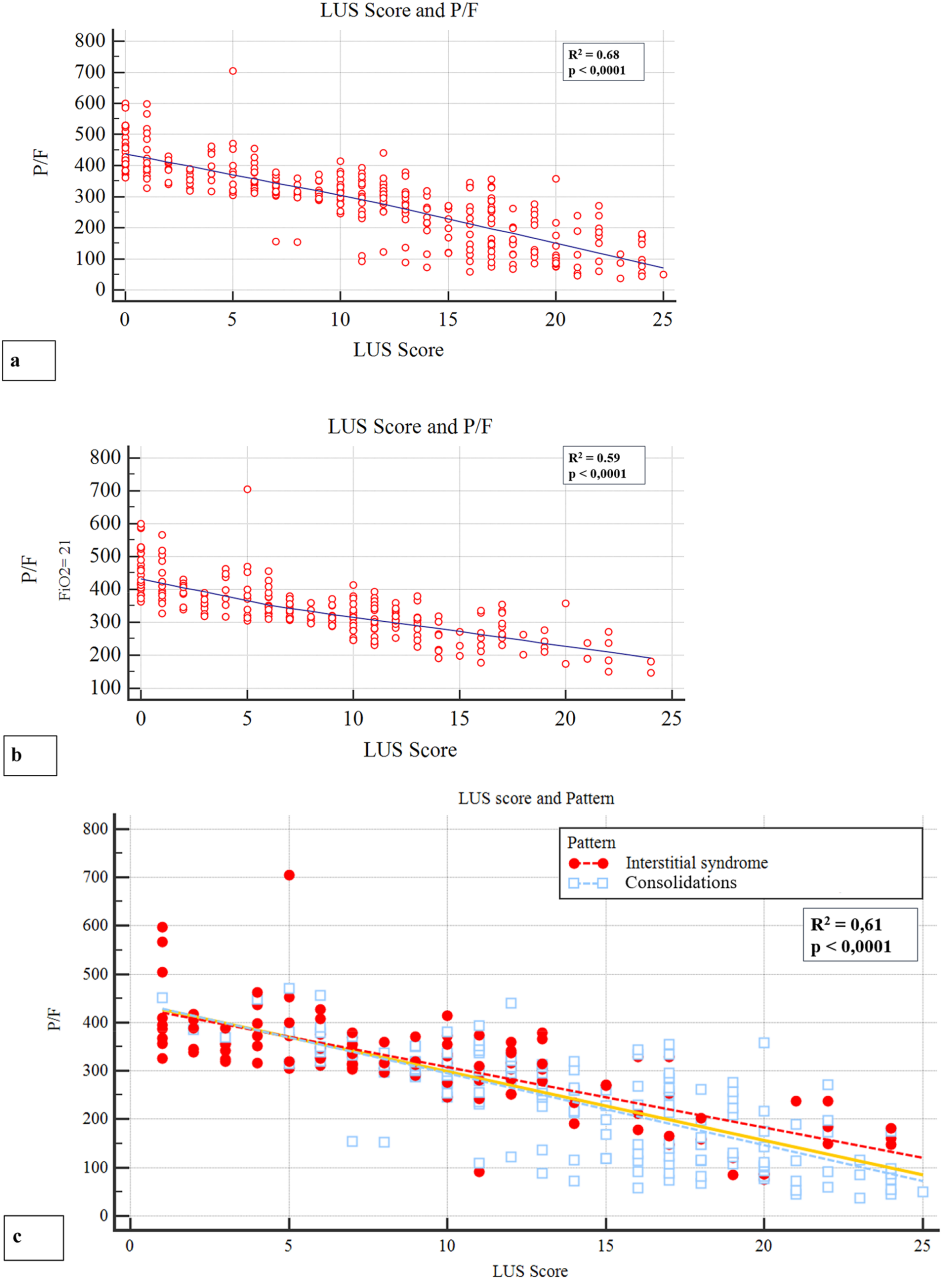
Scatterplots correlation between LUS score and **a** P/F, **b** P/F at FiO_2_ = 21%, **c** correlation between the two different type ultrasound lesions, LUS score, and P/F

**Fig. 4:**
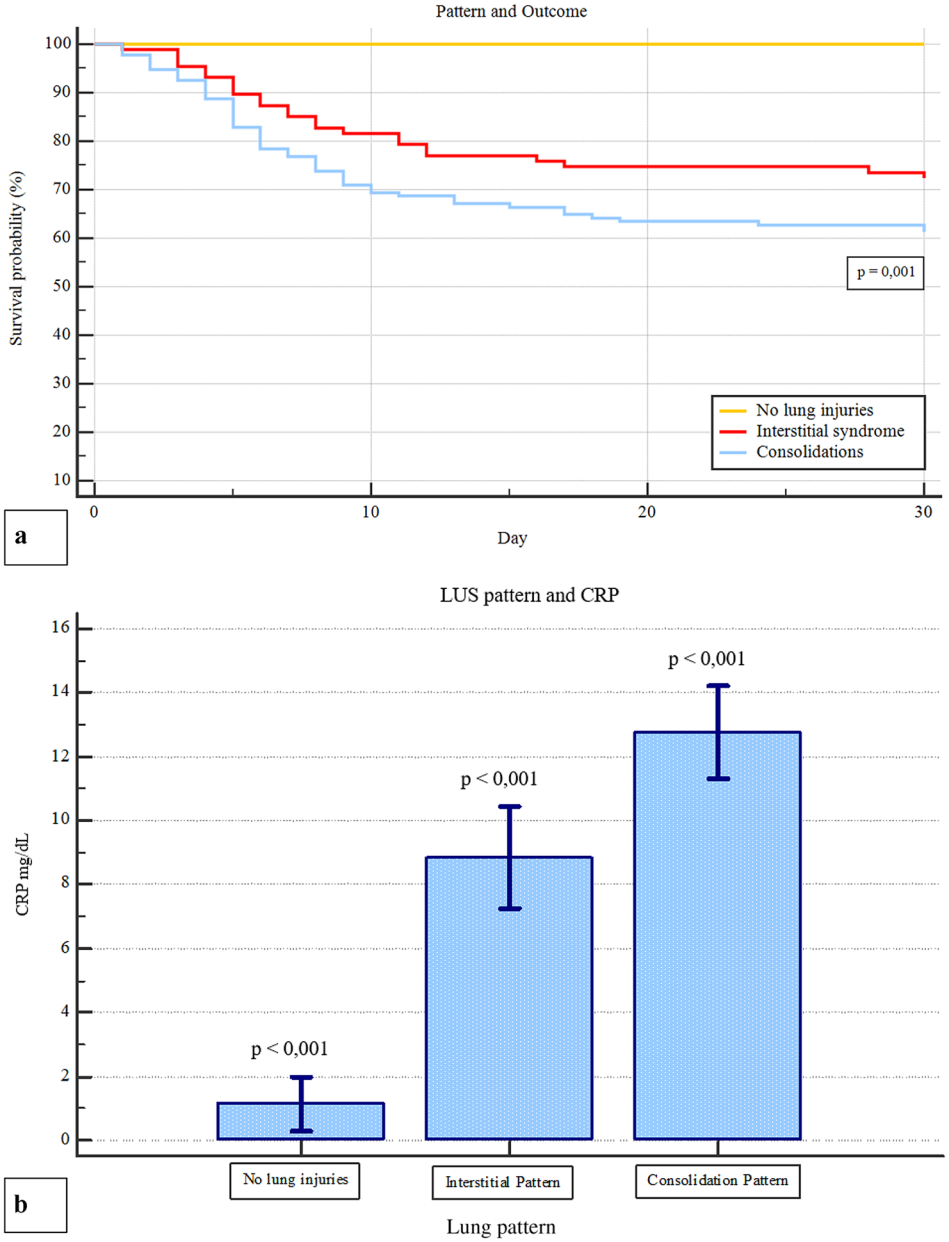
**a** Kaplan–Meier curves of prevalence ultrasound pattern and 30-day survival rate. **b** LUS pattern and CRP

**Fig. 5:**
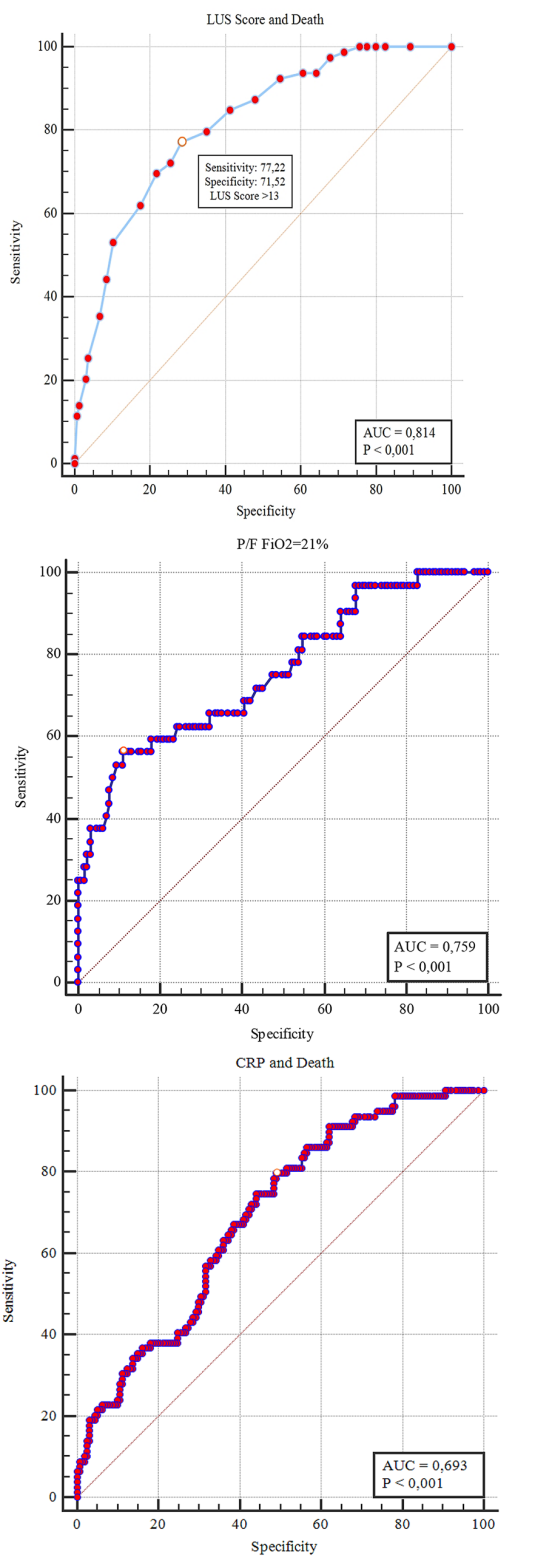
Role of LUS score (top), P/F (middle) and PCR (bottom) in predicting 30-day mortality, according to ROC curve analysis

Only 36/312 (11.5%) patients did not show lung injuries, as defined by a LUS score < 1. Typical ultrasound features included thickening of the pleural line with pleural line irregularities, different vertical artifact (focal, multifocal, or confluent: 87.5%), frequent presence of the “Lightbeam sign” (61.2%) [19], subpleural regional consolidations (51.3%), occasional dynamic air bronchograms and pleural effusion (33.6%). Bilateral lung lesions were evident in 79.5% patients. Upon ED admission, median LUS score was 11 (range 0–25). In general, a much more frequent involvement of the posterior and lateral fields was evident. Topographical distribution of significant lung lesions is summarized in Fig. 2. A tight relationship between LUS score and arterial blood gas was observed, in detail with P/F (*R*^2^ 0.68; *p* < 0.0001), P/F at FiO_2_ on 21% (*R*^2^ 0.59; *p* < 0.0001) (Fig. 3). These correlations underscore the capability of ultrasound imaging in detecting not only structural but also functional derangement of the lungs. As to the prevalent lung ultrasound pattern, 116 (37.1%) patients presented only interstitial syndrome and 160 (51.3%) a consolidation pattern. Both patterns were more prevalent in posterior-inferior fields. Median LUS score was 9 ± 6 and 14 ± 5 in the interstitial and consolidation patterns, respectively (*p* < 0.001). Despite different values of LUS score, the relationship between LUS score and P/F was comparable in the two prevalent lung ultrasound patterns (interstitial syndrome vs. subpleural consolidation) (Fig. 3). In particular, both LUS patterns show similar P/F, at comparable LUS score (*R*^2^ 0.61; *p* < 0.0001). As expected, LUS score in the Emergency Department was progressively higher in patients who were subsequently admitted to General Ward (GW) or to Intensive Care Unit (ICU), as compared with patients who were discharged and confined to home-based quarantine (Table 1). Admitted patients were also older, more frequently males, with higher levels of LDH and PCR, lower lymphocyte count and more severe arterial blood gas alterations. Interstitial pattern was present in 51/168 (30.3%) patients admitted in GW and in 12/30 of those who needed ICU (40%), whereas a consolidation pattern was present in 115/168 (68.4%) and 17/30 (56.7%) patients, respectively (three patients were hospitalized, two in GW, and one in ICU, respectively, without pulmonary involvement, for other medical conditions). Another aspect is that the LUS pattern was related with CRP levels, that were higher in patients with a consolidation pattern (12.8 ± 9.3 mg/dL) as compared with those with interstitial pattern (8.8 ± 8.5) or no pulmonary injuries (1.1 ± 2.3) (p < 0.001) (Fig. 4). The endpoint of 30-day mortality was predicted by LUS score, sensitivity and specificity being 77.2% and 71.5%, respectively, for a LUS score > 13 (AUC = 0.814; p < 0.001). To this respect, LUS resulted more performant than CRP and P/F at ambient air (Fig. 5). As evident from Kaplan–Meier curves, survival was also associated with the pattern of LUS presentation (no lung injuries vs interstitial vs. consolidative pattern; *p* = 0.001). (Fig. 4), underlining a potential prognostic role or its utility for a correct intrahospital triage of Covid-19 patients. Indeed, discharged patients had a lower LUS score (almost ever < 7), and no one of them was readmitted or died during the 30-day follow-up.

## Discussion

The main result of the present study is the snapshot of LUS patterns of Covid-19 pneumonia and their functional correlations with P/F and CRP. Furthermore, LUS score upon ER admission predicts 30-day mortality in COVID-19 patients, in a series that, to the best of our knowledge, is at the moment one of the largest reported so far. Worth of mention is the fact that this study, as many others during this pandemic, was conducted in nearly chaotic conditions due to the overwhelming number of patients invading the ED. Typical ultrasound features defined by Volpicelli et al. [19] as an “explosion of multiform vertical artifacts” were very often present. In detail, 87.5% of the study cohort presented vertical artifacts of different morphology [19]. The “Lightbeam sign” defined as broad, lucent, band-shaped, vertical artifact that moves rapidly with sliding, at times creating an “on–off” effect as it appears and disappears from the screen, was present in 61.2% of the overall population [19]. As shown in Fig. 1, percentage distribution of lung ultrasound injuries underlines the predominant distribution in the posterior and lateral fields of the lungs. Moreover, bilateral lung involvement was present in 79.5% of our population. Such a distribution is in agreement with other cohorts of COVID-19 patients [24], as well as with reported data based upon chest radiography [20] or CT scans, showing bilateral lung involvement, multifocal ground-glass opacities, and consolidation in a typical peripheral with a posterior-dependent gradient and more consolidation in the postero-basal regions [21–23]. Chen et al. [25] reported an excellent correlation between CT and LUS, all abnormal CT findings being also detected by LUS. Moreover, these Authors highlighted the correlation between morphological CT and LUS patterns in COVID 19, the presence of LUS confluent vertical artifacts corresponding to the ground glass CT framework, just in the detection of subpleural lung consolidations [25]. It has to be noted that chest X-ray might not be indicative of the disease, as shown in up to 25% of cases in a series of 240 consecutive patients from our center [26]. As expected, the radiological as well as the ultrasound findings show a time-dependent evolution according to the disease stage at the time of scanning. Therefore care should be taken in comparing different imaging findings in different time points of the natural history of the disease. As to the present study cohort, out of 312 patients, 116 (37.1%) were classified as presenting the “interstitial pattern” and 160 (51.3%) the “consolidation pattern”, respectively. Although these patients were different in terms of clinical presentation, symptom severity, LUS score, blood gas analysis, need of general ward/ICU admission, as well as mortality, the two LUS patterns did not differ in terms of relationship between LUS score and P/F. This underscores the informative role of LUS in evaluation lung structure and function. In a large cohort of swab-positive COVID-19 patients, the present study demostrates that LUS is a powerful diagnostic tool, not only because it facilitates patient rule-in, but also because it helps the early identification of patient’s oxygen need. According with the recent literature, [10] we found a strong correlation with P/F, and with P/F and PO_2_ at a FiO_2_ of 21%, suggesting a correlation with the severity of lung injury. The inverse relationship between P/F ratio and LUS score is a confirmation of the important role of ultrasound evaluation in diagnosing and defining the severity of pneumonia. Observations by different experts as Gattinoni [5, 7] and Volpicelli [7, 19] reports the hypothesis that, at variance with what observed in ARDS, the interstitial and the consolidation patterns equally contribute to the reduction of lung aeration, and that probably it is the overall proportion of lung tissue showing ground glass alterations to determine the severity of respiratory impairment. The correlation we found between P/F and LUS score supports the hypothesis that the severity of respiratory impairment is attributable more to a quantitative than to the qualitative aspect. Furthermore, as shown in Fig. 3, despite different LUS score and survival, the “interstitial syndrome” and the “consolidation” patterns were superimposable in the relationship between semiquantitiative lung involvement and P/F ratio. This adds on the recent hypothesis that in COVID-19 different types of lung injury might equally contribute to the reduction of lung aeration, as proposed by Gattinoni and coworkers [5, 7, 8]. Based on these considerations, it is interesting to note that patients with consolidation pattern have a worse 30-day prognosis (Fig. 3), probably because they have a more advanced stage of lung severity injuries, as demonstrated by the correlation with higher CRP levels when compared with patients with only interstitial syndrome and with those without pulmonary involvement (Fig. 3). Under this respect, LUS may contribute to patient stratification, allowing a bedside distinction between the two patterns. This adds on the other many recognized advantages of LUS, such as portability, bedside evaluation, the lack of need of moving the patient to the radiology department, with subsequent exposure of further healthcare personnel to the risk of COVID-19 infection, as well as repeatability. The latter may allow a serial day-by-day evaluation of disease evaluation and help characterizing the natural history of the disease. It is important to note that LUS score predicts 30-day mortality, as evident from Fig. 5. To the best of our knowledge, little information is present in terms of LUS prognostic role in this setting. The advantage of a bedside tool like ultrasound evaluation in assessing not only the presence of structural and functional alterations, but also in being related to P/F and, even more importantly in predicting mortality cannot be overlooked. LUS gives to the ED healthcare personnel the possibility of diagnosing and stratifying patient’s prognosis since the very first observation, when waiting for the results of the lab testing. Moreover, LUS has the advantages of its intrinsic speed of acquisition, and its being relatively unaffected by the patient's cooperation, that may limit her/his breath-hold capability [27, 28]. A potential limitation is a lower diagnostic power in detecting deeper lesions, that might obviously benefit of radiological/CT evaluation. This appears especially true for deeper foci of pneumonia that do not extend to the pleural surface. Another limitation is related to the lower capability of identifying embolic lesions, as well with the risk of overdiagnosing in the setting of a major outbreak, such as the current one. Despite these limitation, these results indicate that lung ultrasonography has major utility for point-of-care management of COVID-19 pneumonia. As to the method of quantifying LUS score, preliminary reports in COVID-19 era suggest a correlation of LUS findings to those of the CT scan [29, 30]. Soldati et al. [31] have proposed a standardized approach to performing LUS in these patients, including a 14-zone technique, and a scoring system to quantify the severity of lung involvement. Undoubtely a wider consensus is needed. In the present study, we restricted our study to a 12-zone approach, that is faster in Emergency setting, and that was validated with CT findings [32]. Future studies are needed to compare these different methods and to extend the analysis to a 14-zone approach. Whatever the tecnhique, these results indicate an important role of LUS score in the evaluation of COVID-19 pneumonia in the Emergency Department. It requires less time than RT-PCR results, and when properly associated with the clinical evaluation it can rapidly direct “rule-in” and “rule-out” of the patients, as well as their intra-hospital triage. Given the many advantages of LUS and the availability of portable instruments, it might also be hypothesized a role of this imaging technique in the out-of-hospital evaluation of patients, that appears particularly important in the current pandemics as a first-aid tool to triage patients already in the field setting [33]. Further studies are needed to explore LUS patterns during the entire disease course.

## Conclusion

Bedside LUS represents an effective and fast tool for both the diagnosis definition and the prognostic stratification of COVID-19 pneumonia in the Emergency Department. LUS may be used in the ER to early identify COVID-19 worst patients and to correctly triage those patients with more extensive lung involvement who should be admitted to the General Ward or Intensive Care Unit (ICU). The study supports the primacy of LUS as the "go to" imaging modality for initial and ongoing management of COVID-19 respiratory failure leaving the indication of chest CT scan as reserved only for the more complex cases. Therefore, LUS routine integration into the clinical management of this challenging disease is strongly suggested.
